# High Seroprevalence of Anti-SARS-CoV-2 Antibodies in Children in Vietnam: An Observational, Hospital-Based Study

**DOI:** 10.3390/pathogens11121442

**Published:** 2022-11-30

**Authors:** Dien Minh Tran, Uyen Tu Thi Vu, Canh Ngoc Hoang, Ha Thu Thi Nguyen, Phu Huy Nguyen, Mai Chi Thi Tran, Anh Ngoc Chu, Phuc Huu Phan

**Affiliations:** 1 Surgical Intensive Care Unit, Vietnam National Children’s Hospital, 18/879 Lathanh Street, Hanoi 100000, Vietnam; 2 Department of Biochemistry, Vietnam National Children’s Hospital, 18/879 Lathanh Street, Hanoi 100000, Vietnam; 3 Pediatric Intensive Care Unit, Vietnam National Children’s Hospital, 18/879 Lathanh Street, Hanoi 100000, Vietnam; 4 Department of Immunology, Allergy, and Rheumatology, Vietnam National Children’s Hospital, 18/879 Lathanh Street, Hanoi 100000, Vietnam; 5 Research Office, Vietnam National Children’s Hospital, 18/879 Lathanh Street, Hanoi 100000, Vietnam

**Keywords:** children, seroprevalence, antibodies, SARS-CoV-2, Vietnam

## Abstract

**Background:** The robustness of sero-surveillance has delineated the high burden of SARS-CoV-2 infection in children; however, these existing data showed wide variation. This study aimed to identify the serostatus of antibodies against SARS-CoV-2 and associated factors among children following the fourth pandemic wave in Vietnam. **Methods:** A cross-sectional study was conducted at Vietnam National Children’s Hospital (VNCH) between March 13 and April 3, 2022. Thus, 4032 eligible children seeking medical care for any medical condition not related to acute COVID-19 infection were tested for IgG SARS-CoV-2 antibodies by *ADVIA Centaur*^®^ SARS-CoV-2 IgG (*sCOVG*) *assay* using the residuals of routine blood samples. **Results:** The median age of enrolled children was 39 (IQR = 14–82) months. The overall seropositive prevalence was 59.2% (95%CI = 57.6–60.7) and the median antibody titer was 4.78 (IQR 2.38–9.57) UI/mL. The risk of seropositivity and the median antibody titer were not related to gender (58.6% versus 60.1%, 4.9 versus 4.6 UI/mL, all *p* > 0.05). Children aged ≤12 months were likely to be seropositive compared to children aged 36 to <60 months (59.2% versus 57.5%, *p* = 0.49) and those aged ≥144 months (59.2% versus 65.5%, *p* = 0.16). Children aged ≥144 months exhibited a significantly higher titer of protective COVID-19 antibodies than other age groups (*p* < 0.001). In multivariate logistic regression, we observed independent factors associated with SARS-CoV-2 seropositivity, including the age 13 to <36 months (OR = 1.29, 95%CI = 1.06–1.56, *p* = 0.01), 60 to <144 months (OR = 0.79, 95%CI = 0.67–0.95, *p* = 0.01), ≥144 months (OR = 1.84, 95%CI = 1.21–2.8, *p* = 0.005), the presence of infected household members (OR = 2.36, 95%CI = 2.06–2.70, *p* < 0.001), participants from Hanoi (OR = 1.54, 95%CI = 1.34–1.77, *p* < 0.001), underlying conditions (OR = 0.71, 95%CI = 0.60–0.85, *p* ≤ 0.001), and using corticosteroids or immunosuppressants (OR = 0.64, 95%CI = 0.48–0.86, *p* = 0.003). **Conclusions:** This study highlights a high seroprevalence of antibodies against SARS-CoV-2 among children seeking medical care for non-acute COVID-19-related conditions in a tertiary children’s hospital in Hanoi, Vietnam. In the context of reopening in-person schools and future emerging COVID-19 variants, this point will also be a key message about the necessity of “rush-out” immunization coverage for children, especially those under the age of five years.

## 1. Introduction

Since the first report of novel coronavirus infection in the Hubei province of China in late 2019, the Coronavirus disease 2019 (COVID-19) has now been known as a global public-health catastrophe, with significant mortality and morbidity [1]. According to the latest data from the Center for Systems Science and Engineering (CSSE) at Johns Hopkins University (JHU), SARS-CoV-2 has infected approximately 625,344,144 people, causing more than 6.5 million deaths (https://coronavirus.jhu.edu/map.html accessed on 18 October 2022).

Like other viral respiratory infections, the novel coronavirus can be transmitted through the respiratory tract, manifesting a broad spectrum of clinical manifestations, from asymptomatic to severe respiratory failure, multiorgan dysfunctions, and death [2,3]. COVID-19, also called severe acute respiratory syndrome coronavirus 2 (SARS-CoV-2), is known to affect adults predominantly with greater risks of severe conditions in vulnerable individuals, such as immunocompromised, pre-existing comorbidities, and older people [2,4,5,6,7]. However, data regarding the burden of COVID-19 in children remain limited. Of note, earlier data suggested that children are less likely to contract the virus than adults [8,9,10,11]. Recent studies have shown that children had a similar prevalence of SARS-CoV-2 infections compared to adults but majorly presented in the asymptomatic form; therefore, many of those were not tested at all [12]. This potentially leads to an underestimated burden of infections, in which the actual number of infected people is substantially higher than the reported cases [13,14]. Remarkably, asymptomatic cases might have a critical role in transmission and infected children remain at risk of severe complications, including hospitalization, multisystem inflammatory syndrome, post-COVID-19 conditions, and death [15,16]. Hence, the ongoing surveillance of the real burden of infections in pediatrics is vital for guiding political decisions to prevent pandemic expansion, counting school closures, and vaccination strategies.

The reverse-transcription polymerase chain reaction (RT-PCR) test using upper respiratory specimens is considered the gold standard for confirming SARS-CoV-2 infection [17]. Nevertheless, low sensitivity of the RT-PCR test may induce false-negative SARS-CoV-2 results, widely ranging from 1% to 30% due to several factors, such as insufficient samples, inappropriate specimen type, the timing of testing since exposure, and low viral load [17,18,19]. Interestingly, unlike typical seroconversion profiles, near-simultaneous production of both immunoglobulin-M (IgM) and immunoglobulin-G (IgG) was observed after approximately 1–3 weeks of both symptomatic and asymptomatic COVID-19 infection [20,21]. IgM eventually disappears after 4 to 6 weeks of infection, while IgG remains detectable within six months afterward [22,23]. Therefore, a novel approach focusing on detecting SARS-CoV-2 anti-nucleocapsid and anti-spike protein IgG might be a potential method for a more accurate estimation of asymptomatic or subclinical infections for several months after infection [14,19]. Various subsequent studies on the seroprevalence of SARS-CoV-2 among children have been carried out worldwide but enunciated a wide range of positivity rates, depending on studies, timeframe, and geography [12,14,19,24,25]; therefore, additional studies are required.

Spiking COVID-19 infection among children during the Omicron variant surge and in the new-normal period has driven an increasing number of vaccines being authorized for use in children of 6 months and above (fda.gov accessed on 17 June 2022). However, many countries, including Vietnam, have given emergency-use vaccination only for children aged five and older; children aged six months and below remain out of protection. It is clear that the decision to immunize children, indeed, accounts for the safety and effectiveness of vaccines and prioritization to the highest-risk age groups; however, it is also necessary to synchronously consider each country’s specific epidemiological and social context to develop their relevant COVID-19 immunization policies and programs. Therefore, this study aimed to identify the serostatus of antibodies against SARS-CoV-2 and associated risk factors in children who seek medical-care unrelated to COVID-19 in a tertiary hospital in Vietnam. There are also available data providing timely recommendations for establishing national immunization campaigns for children under five years, particularly children below six months, who stay on the front line of being vulnerable to COVID-19 infection.

## 2. Materials and Methods

### 2.1. Study Design and Patients

This cross-sectional study was conducted in Vietnam National Children’s Hospital (VNCH) between 13 March and 3 April 2022. The study was approved by the Ethics Committee for Biomedical Research of the hospital (Approval no. VNCH-TRICH-2022-2A) and registered at clinicaltrials.gov (trial registration: NCT05358626) on 13 March 2022.

In-patient or outpatient children aged from 0 to 18 years old seeking medical care at Vietnam National Children’s Hospital for any condition unrelated to acute COVID-19 were eligible to participate. For both groups, additional eligibility criteria were having residual blood specimens collected from routine biochemistry tests. Patients with positive antigen tests for SARS-CoV-2 at enrollment, those who received COVID-19 inoculation within six months, those with blood samples stored over 24 h or not enough residual blood (at least 2 mL), or those with refused via their guardians to participate in this study were excluded.

### 2.2. Data Collection

Data were collected using standard case report forms (CRFs) through in-person interviews with parents/caregivers during medical examination or treatment. The interviews were conducted by research team members experienced and qualified in data collection. We obtained verbal consent from the guardians of all participants. Details of the questionnaire included: i. general information (full name, age, gender, demographics, ethnicity); ii. personal and family history (gestational age, birth weight, underlying disease, COVID-19 vaccination record, history of COVID-19 infection, blood transfusion or immunoglobulin infusion within the last six months, history of using corticosteroids or immunosuppressants); iii. maternal history for the child aged equal or less than 12 months old during pregnancy/labor period (COVID-19 vaccination record, history of COVID-19 infection, blood transfusion or immunoglobulin infusion, breast-feeding); iv. laboratory variables (qualitative status: seropositive/seronegative, titer level of IgG against SARS-CoV-2: UI/mL). The primary outcomes were the seroprevalence and mean titer of protective antibodies against SARS-CoV-2. The secondary outcomes were the association between serostatus of anti-SARS-CoV-2 antibodies and socio-demographic characteristics and personal and household history.

### 2.3. Serological Samples and Laboratory Procedure

Samples of residual heparinized blood from routine biochemistry tests were centrifuged for 5 min at 3000 revolutions per minute. These samples werr pipetted into an Eppendorf cup and then stored at −20 °C until analysis. The ADVIA Centaur (sCOVG) assays are processed using an ADVIA Centaur XP system from Siemens Healthcare Diagnostics system. These assays were run fully simultaneously with a two-step sandwich immunoassay using indirect chemiluminescent technology. The patient specimens were diluted with ADVIA Centaur sCOVG DIL and incubated with the Solid-Phase Reagent, forming a preformed complex of streptavidin-coated microparticles and biotinylated SARS-CoV-2 recombinant antigens. Subsequently, the antibody–antigen complex stemed from SARS-CoV-2-specific antibodies after capturing the antigen-coated particles in the specimen. The antibody–antigen complex was washed before adding Lite Reagent, which comprises an acridinium-ester-labeled anti-human IgG mouse monoclonal antibody. After washing the whole complex, the serostatus was interpreted through the presence of Lite Reagent binding to the Solid Phase via the anti-SARS-CoV-2 IgG:SARS-CoV-2 antigen complex. Based on the amount of relative light units (RLUs) detected by the system, the SARS-CoV-2 IgG antibody level was calculated in units per milliliter (UI/mL). The result interpretation was as follows: i. nonreactive: <1.00 Index (UI/mL)—these samples were considered negative for SARS-CoV-2 IgG antibodies; ii. reactive: ≥1.00 Index (UI/mL)—these samples were considered positive for SARS-CoV-2 IgG antibodies [26]. The Siemens assay was demonstrated to be a reliable serological test to detect total anti-SARS-CoV-2 antibodies that provide high sensitivity and specificity. The antibody levels significantly increased from the day of the RT-PCR diagnosis and symptomatic onset, reaching 96.41% (95%CI = 92.74%–98.54%) and 97.78% (95%CI = 92.26–99.39) [26] in the third week, respectively. Similarly, the clinical specificity for the assay was also high, leading to 99.99% (95%CI = 99.64%–99.99%) [26], which makes the general performance of the assay accurate for political decision-making in pandemic management.

### 2.4. Statistical Analysis

All statistical analyses were performed using STATA software version 17.0 (StataCorp LP, College Station, TX, USA). Categorical variables were described as frequencies and percentages, while continuous variables were described as the median and interquartile range. We used the Mann–Whitney U test and the Kruskal–Wallis test to compare the medians of anti-SARS-CoV-2 IgG level among independent subgroups. Comparison of seropositive rates was performed by Chi-square test or Fisher’s Exact test. Bivariate analyses of SARS-CoV-2 seroprevalence were performed according to age, gender, demographics, underlying conditions, type of patient care, personal and family history of COVID-19 infection and vaccination, and history using corticosteroids or immunosuppressants. Results were described as odds ratio (OR) and 95% confidence intervals (CIs). Variables significantly associated with SARS-CoV-2 seropositivity in univariate analyses were selected for multivariate logistic progression to identify independent factors of SARS-CoV-2 seropositivity. A two-sided *p*-value of less than 0.05 was considered statistically significant.

## 3. Results

**Figure 1:**
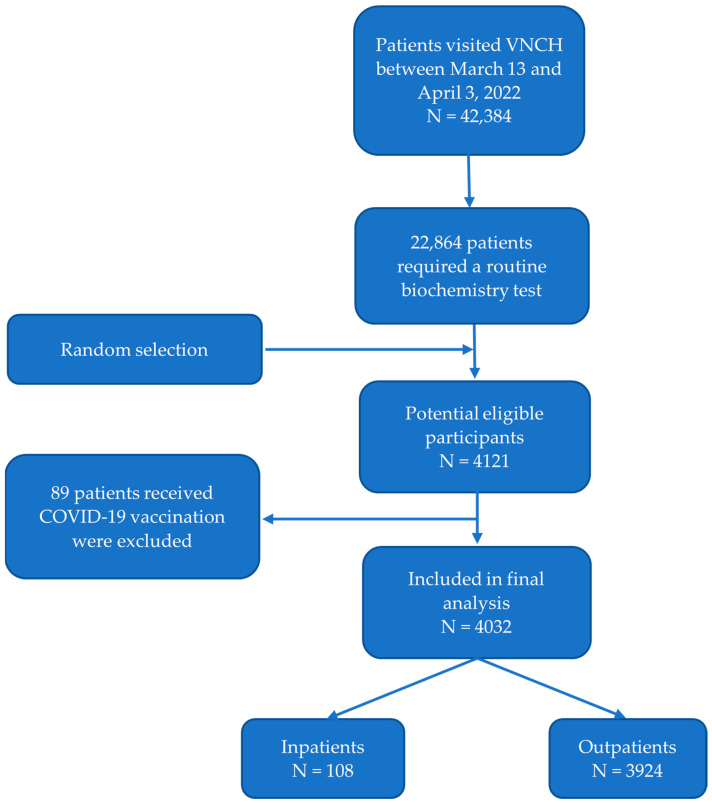
Flow diagram of study participants.

During the study period, 42,384 patients visited Vietnam National Children’s Hospital, of whom 22,864 patients required a biochemistry test. Residual specimens of 4121 patients were randomly collected to test for IgG antibodies against SARS-CoV-2. We excluded 89 patients receiving COVID-19 vaccine shots. Therefore, the remaining 4032 patients, including 108 inpatients and 3924 outpatients, were eligible for final analysis (Figure 1).

### 3.1. Patient Characteristics

We enrolled 4032 patients with a median age of 39 (IQR = 14–82) months, of whom 2447 (60.7%) were male. The other patient characteristics are presented in Table 1.

### 3.2. Seroprevalence and Titer Level of Anti-SARS-CoV-2 Antibodies

Thus, 2385 out of 4032 participants had a positive test for anti-SARS-CoV-2 antibodies, corresponding to an overall seroprevalence of 59.2% (95% CI 57.6–60.7). The median antibody titer level of the seropositive subgroup was 4.78 (IQR 2.38–9.57) UI/mL (Table 1).

### 3.3. Factors Associated with Anti-SARS-CoV-2 Antibody Seropositivity and Antibody Titer Level

We assessed the quantitative and qualitative difference in anti-SARS-CoV-2 antibodies on multiple variables of socio-demographic characteristics and personal and family history—age, gender, gestational age, residence, underlying disease, type of patient care, history of COVID-19 infection, history of immunoglobin or blood transfusion, and history of using corticosteroids or immunosuppressants (Table 2 and Table 3).

#### 3.3.1. Gender, Types of Care, and History of Prematurity

The rates of seropositivity and titer level were not influenced by gender, different types of care, and history of prematurity (all *p* > 0.05) (Table 2 and Table 3).

#### 3.3.2. Age

The seroprevalence varied according to age group: Children aged ≤ 12 months were likely to be seropositive compared to children aged 36 to <60 months (59.2% versus 57.5%, *p* = 0.49) and those aged ≥ 144 months (59.2% versus 65.5%, *p* = 0.16) (Table 2). Children aged ≥ 144 months exhibited a significantly higher titer of protective COVID-19 antibodies compared to other age groups (*p* < 0.001) (Table 3)**.**

#### 3.3.3. Presence of Infected Household Members

We found evidence for clustering infections within households; participants with at least one infected household member were more likely to be seropositive than those without an infected household member (65.8% vs. 48.5%, OR = 2.04, 95%CI = 1.78–2.33, *p* = 0.001) (Table 2).

#### 3.3.4. Residences

Participants from Hanoi were at higher risk of being seropositive than those from other provinces (62.6% vs. 53.3%, OR = 1.46, 95%CI = 1.29–1.67, *p* = 0.0001) (Table 2). However, the level titer of SARS-CoV-2 antibodies was not influenced by locations of residence.

#### 3.3.5. History of Underlying or Using Corticosteroids or Immunosuppressants

Our results also showed that patients without underlying conditions or using corticosteroids or immunosuppressants had significantly higher seroprevalence and titer levels of SARS-CoV-2 antibodies than their counterparts, respectively (Table 2 and Table 3).

Our result recognized age group of 13 to <36 months and ≥144 months, residence from Hanoi, and presence of COVID-19 infection of household members as independent factors positively related to SARS-CoV-2 seropositivity. In contrast, seropositivity for anti-SARS-CoV-2 antibody was conversely associated to the presence of underlying conditions and history of using corticosteroids or immunosuppressants (Table 4).

#### 3.3.6. Infants Aged ≤ 12 Months Old

For infants aged ≤12 months old, maternal history during pregnancy/labor period might surrogate hazard of anti-SARS-CoV-2 antibody status; thus, we investigated the influence of these factors on IgG seropositivity and titer level. Of those, children with a maternal history of COVID-19 infection showed higher antibody prevalence and titer levels than their corresponding group, with 70.6% and 53.8%, respectively (OR = 2.06, *p* < 0.05). However, maternal history of COVID-19 vaccination and breastfeeding was not associated with antibody state (Table 5).

Among 939 mothers of infants aged ≤ 12 months, 316 (33.6%) reported having COVID-19 infection during pregnancy/labor period, of whom 313 mothers remember the time of past infection, including 1 (0.3%) case before pregnancy; 25 (8.0%) cases during pregnancy; and 287 (91.7%) cases after delivery. For 25 mothers infected during pregnancy, the number of infections in the second trimester, third trimester, and during labor was 1, 23, and 1, respectively. 

We recorded 456 out of 939 mothers as having received vaccination during this period, including 77 cases (16.9%) receiving only one shot, 259 cases (56.8%) receiving two shots, and 120 (26.3%) receiving three shots. Among these 456 cases, there were 13 (2.9%), 85 (18.6%) and 358 (78.5%) cases received their first vaccination before pregnancy, during pregnancy, and after delivery, respectively.

## 4. Discussion

This paper reported a sero-surveillance of anti-SARS-CoV-2 antibodies conducted on 4032 children seeking medical care in a tertiary children’s hospital in Vietnam between 13 March and 3 April 2022. Our findings highlight a substantially high seropositivity rate of IgG against SARS-CoV-2 in the studied population and raise questions about the real burden of COVID-19 on children. Additionally, we found that the hazard of SARS-CoV-2 seropositivity was considerably associated with the clustering of infections within households, geography, underlying conditions, and history of using corticosteroids or immunosuppressants. This paper provided a snapshot of the hidden burden of susceptibility to SARS-CoV-2 in the pediatric population, which expressed the need for rush-out vaccination coverage in children, particularly those under five years, fitting the context of school reopening and future emerging variants.

Earlier data about COVID-19 suggested that children are less likely to contract and have a much milder course of infection than adults [8,9,10,11], which is supported by several generated hypotheses. These potential explanations were extensively discussed from insight into specific properties of children, comprising the lower expression in receptors of angiotensin-converting enzyme 2 (ACE2), cross-protection against SARS-CoV-2 infection, and their immune system response [13,27]. With human-to-human transmitting pathways of COVID-19, given that most pediatric cases were described inside familial clusters, another theory is that children are less likely to be exposed to the virus due to fewer community interactions and outdoor activities than adults [7,27,28]. This is in line with several early studies from different places, such as Iceland, Toulouse, Geneva, Austria, and Wuhan/Shanghai, where the percentage of seropositive children was significantly lower than that of adults [12,29,30,31,32]. However, recent studies have shown that children had a similar prevalence of SARS-CoV-2 infections compared to adults but presented in the asymptomatic form in a majority; many of those were not tested at all [12]. Recently, reports from previous sero-surveillance yielded a high seroprevalence of asymptomatic status [19,33], causing a misleading exact burden of COVID-19 infection in children.

To the best of our knowledge, the first published sero-surveillance for anti-SARS-CoV-2 antibodies in May 2020 reported a wide range of positive estimates, from 0.4 to 59.3% of the general population [24]. Various subsequent studies have been carried out worldwide; however, available data on the pediatric seroprevalence for anti-SARS-CoV-2 antibodies enunciates a high uncertainty. Remarkably, seroprevalence considerably varied among studies, timeframe, and geography [12,14,19,25]. Our results also indicated that 2385 of 4032 patients were seropositive, corresponding to a high overall seroprevalence (59.2%) of immunity against SARS-CoV-2 (Table 1). Our seropositivity rate was significantly higher than that of previous studies, such as in Canada (5.8%) [34], the United Kingdom (6.9%) [19], and Croatia (2.9–8.4%) [33], which were mainly conducted during the second wave of the pandemic. In our context, during the fourth wave of COVID-19, in which the dominant Omicron variant had higher transmissibility than the original variants, the high seroprevalence was consistent with the more rapid increased daily case incidence [35]. Similarly, serologic data at the timeframe of Omicron variant predominance in South Africa showed that both seroprevalences of children 12 to 17 years of age and those younger than 12 years of age were high, with 73.8% and 56.2%, respectively [36]. The younger children are, the more they frequently depend on their caregivers and are exposed to the potential risk of exposure to pathogens; not to mention, this population presented asymptomatically in the majority, which is likely contributable to a “chain joint” in viral transmission over time. Additionally, most children with multisystem inflammatory syndrome (MIS-C) are between the ages of 3 and 12 years old, with an average age of 8 to 9 years old [37]. Consequently, the U.S. Food and Drug Administration (FDA) recently authorized the emergency use of the Moderna and the Pfizer-BioNTech COVID-19 Vaccine for the prevention of COVID-19 to include children down to 6 months of age (fda.gov accessed on 17 June 2022). Despite the latest data about the safety and effectiveness of COVID-19 vaccines on mitigating future contraction rates, hospitalizations, long-term influence, and deaths in children [38,39,40,41,42], worldwide officials raise controversies around immunization benefits and vaccine safety in such age groups, particularly children under five years. For many countries, including Vietnam, the vaccines were approved only for children aged five and older, while few countries, such as the United States, Brazil, and Costa Rica, have vaccinated children from six months old [38]. Our results showed that children aged ≤ 12 months were likely equal to be seropositive compared to children aged 36 to 60 months (59.2% vs. 57.5%, OR = 0.93, 95%CI = 0.75–1.14, *p* = 0.49) and those aged ≥ 144 months (59.2% vs. 65.5%, OR = 1.31, 95%CI = 0.87–1.96, *p* = 0.16) (Table 2). Remarkably, our results expressed that the highest seroprevalence rate was reported in children aged 13 to <36 months old (Table 2), who currently remain out of vaccination coverage in Vietnam and most countries. Given the potential ongoing reservations and sources of newly emerged variants [39], we reinforce that a “rush-out” national immunization for the pediatric population, particularly children aged less than three years, should begin now. Basically, the decision to immunize children, indeed, accounts for the safety and effectiveness of vaccines and prioritization to the highest-risk age groups; however, it is also necessary to synchronously consider each country’s specific epidemiological and social context to develop their relevant COVID-19 immunization policies and programs.

As expected, there was considerable variation in the seroprevalence of anti-SAR-CoV-2 antibodies according to geographics. Hanoi capital had significantly higher seroprevalence than all other provinces, at 62.6% and 53.3% (OR = 1.46, 95%CI = 1.29–1.67, *p* = 0.0001) (Table 2). This point complied with the densely populated urban areas of Hanoi, regarded as the epicenter of the COVID-19 pandemic in Vietnam for six months prior to our study period. Of note, local epidemiological data are crucial for guidance on preventing SARS-CoV-2 pandemic expansion, such as the priority of vaccination roll-out to these high-risk regions.

Our results confirm that the seroprevalence of the group with at least one household member presenting COVID-19 infection was higher than their counterpart (65.8% vs. 48.5%, OR = 2.04, 95%CI = 1.79–2.33, *p* = 0.0001) (Table 2), in accordance with other studies [7,27,43]. Young children tend to depend significantly on their caregivers, so appropriate quarantine and separation from other household members can be particularly challenging in this population. Although the early closing of all schools, ranging from kindergarten to universities, might reduce the number of social contacts, the potential transmission of the virus still exists in a confined space [43]. According to Wu et al., most children contract the SARS-CoV-2 virus during contact with family members (85.2%) instead of the community [44]. Another study noted an uncommon correlation between outbreaks in educational settings and the incidence of COVID-19 in the community [45]. These findings might provide benchmark worldwide debates around the true efficacy of school closures in minimizing the SAR-CoV-2 burden in possible future outbreaks. Hence, now more than ever, the ongoing vaccination campaigns in this new-normal period will play a key role in the opportunity for sustaining safe in-person schooling, extracurricular activities, and sports for children.

Interestingly, our results also showed that patients without underlying conditions or without using corticosteroids or immunosuppressants had significantly higher seroprevalence and protective titer levels of SARS-CoV-2 antibodies than their counterparts (Table 2 and Table 3). This is possibly explained by the fact that the immunocompromised are vulnerable individuals with a higher risk of infection, hospitalization, and mortality [46]; therefore, they tend to comply precautiously with the preventive measures to alleviate the risk of infection. Additionally, we provoke hypotheses that if the immunogenicity of both groups is comparable, should a serological test be utilized to determine the exact incidence of infection in the immunocompromised population? A sparse number of studies verified that immunosuppressive adults have effective immunogenicity-producing antibodies to SARS-CoV-2 infection, though a slightly delayed response compared to the immunocompetence [47]. However, these data on the pediatric population were not well understood and required further investigation.

Seroprevalence in children under 12 months old also depends on several factors, including vertically transferred immunity with a maternal history of COVID-19 vaccination or infection or breastfeeding, misinterpreting the actual burden of SARS-CoV-2 in this population [48]. However, previous data showed that vertical transmission rates of SARS-CoV-2 during pregnancy are low, estimated at around 2–3% [49], inducing an insignificant change in fetal seropositivity. Our result also showed that the history of COVID-19 vaccination and breastfeeding was not associated with the hazard of seropositivity (Table 5). However, as presented in Table 5, infants aged ≤ 12 months old with a maternal history of COVID-19 infection during the pregnancy/labor period were more likely to be seropositive than their counterparts. This finding could be explained by the indispensable dependence of children aged 12 months and younger on their caregivers, accumulating more potential risk of seropositivity by exposure to pathogens from their infected mothers after birth. In line with this point, our result showed that among 316 mothers who reported having COVID-19 infection during the pregnancy/labor period, the majority of infected mothers (287 cases, 91.7%) recorded after delivery, while only 1 (0.3%) case and 25 (8.0%) cases were infected before pregnancy and during pregnancy, respectively. Another potential cause that may play a role in transmitting SARS-CoV-2 infection from the mother to the fetus is vaginal delivery [50]. Several studies also suggested the possibility of viral transmission through the placenta and breastfeeding; nonetheless, the evidence on that issue has not been fully understood [49]. We expressed that the differences in the ability of robust immunity responses to SARS-CoV-2 and the sustaining of long-lasting immunity between infants under 12 months and other age groups should be clarified in further studies.

Our study also has some limitations. First, the patients were recruited from a single center in a tertiary hospital, which is not fully representative of all settings in Vietnam and other countries. However, as the largest referral tertiary children’s hospital in the north of the country, patients visiting the hospital were from different geographic regions. Second, IgG is only detectable within six months of infection afterward and reflected approximately estimated cases of COVID-19 infections during the previous six-month period. The timeframe in our study regarding the context of a new wave of the Omicron BA.2 variant that emerged in Hanoi, Vietnam, was during late 2021 and early 2022; thus, the data could not be generalized for different times. Third, due to potential cross-reactivity between SARS-CoV-2 antigens and seasonal coronaviruses [51], false-positive results for the assay may occur, and our seroprevalence rates might be overestimated. Additionally, testing performance may vary depending on the variants circulating [26], such as newly emerging strains of the Omicron compared to original variants. Fourth, missing the clinical manifestations and biochemical data in most cases is a limitation in our study, so that we could not analyze their relationship with other factors. Due to a lack of data on the specific numbers of contaminated members in households, we could not compare the risk of seropositivity between clusters with different numbers of infected family members. Hence, further investigations involving well-designed studies are needed to clarify this issue. Despite limitations, there is an extensive sample size in this study about the seroprevalence of anti-SARS-CoV-2 antibodies in children to the best of our knowledge. This paper provided a snapshot of a high burden of COVID-19 infection in the pediatric population, those generally presenting with mild or asymptomatic infections. In the meantime, this finding is a benchmark, supporting public-health providers and policymakers to roll out appropriate disease-management strategies.

## 5. Conclusions

Our study highlights a high seroprevalence of anti-SARS-CoV-2 antibodies among children seeking medical care for non-acute COVID-19-related conditions in a tertiary hospital in Hanoi, Vietnam. In the context of reopening in-person schools and future emerging COVID-19 variants, this point will also be a key message about the necessity of “rush-out” immunization coverage for children, especially those under the age of five years.

## Figures and Tables

**Table 1:** Patients’ demographics.

Characteristics	Value (N = 4032)
Demographic data	
Age, months *(median, IQR*)	39 (14–82)
Male sex *(n, %)*	2447 (60.7%)
Kinh Ethnic *(n, %)*	3950 (97.9%)
Residences *(n, %)*	
Hanoi	2452 (60.8%)
Other provinces	1580 (39.2%)
Underlying conditions *(n, %)*	
No underlying diseases	3199 (79.1%)
Respiratory system	46 (1.1%)
Cardiovascular system	58 (1.4%)
Gastrointestinal system	92 (2.3%)
Kidney and urology system	117 (2.9%)
Immunodeficiency	14 (0.4%)
Other underlying conditions	516 (12.8%)
Seropositive Prevalence *(n, %)*	2385 (59.2%)
Anti-SARS-CoV-2 antibodies titer level of seropositive subgroup *(UI/mL) (median, IQR)*	4.78 (2.38–9.57)
Receiving blood product or immunoglobulin infusion within 6 months *(n, %)*	22 (0.55%)

Data are presented as median (IQR: Q1–Q3) or number (%). IQR: Interquartile Range, COVID-19: Coronavirus disease 2019.

**Table 2:** The difference in seroprevalence of Anti-SARS-CoV-2 antibodies among subgroups of socio-demographic characteristics and personal and family history.

Subgroups	Overall, n (%)	Positive, n (%)	Negative, n (%)	OR (95%CI)	*p*-Value *
**Age (months)**					
*≤12*	939 (23.3%)	556 (59.2%)	283 (40.8%)	1	
*13 to <36*	967 (23.9%)	642 (66.4%)	325 (33.6%)	1.36 (1.13–1.63)	0.001
*36 to <60*	628 (15.6%)	361 (57.5%)	267 (42.5%)	0.93 (0.75–1.14)	0.49
*60 to <144*	1382 (34.3%)	750 (54.3%)	632 (45.7%)	0.82 (0.69–0.97)	*0.02*
*≥144*	116 (2.9%)	76 (65.5%)	40 (34.5%)	1.31 (0.87–1.96)	0.16
**Gender**					
*Male*	2447 (60.7%)	1434 (58.6%)	1013 (41.4%)	0.94 (0.83–1.08)	0.38
*Female*	1585 (39.3%)	951 (60.0%)	634 (40.0%)		
**Residence**					
*Hanoi*	2452 (60.8%)	1540 (62.6%)	912 (37.2%)	1.46 (1.29–1.67)	0.0001
*Other provinces*	1580 (39.2%)	845 (53.3%)	735 (46.5%)		
**Types of patient care**					
*In-patient*	108 (2.7%)	59 (54.6%)	49 (45.5%)	0.83 (0.55–1.24)	0.33
*Out-patient*	3924 (97.3%)	2326 (59.3%)	1598 (40.7%)		
**Previous COVID-19 infection of household members**					
*Yes*	2476 (61.4%)	1630 (65.8%)	846 (34.2%)	2.04 (1.79–2.33)	0.001
*No*	1556 (38.6%)	755 (48.5%)	801 (51.5%)		
**History of using corticosteroids or immunosuppressants**					
*Yes*	242 (6.0%)	103 (42.6%)	139 (57.4%)	0.49 (0.37–0.64)	0.001
*No*	3790 (94.0%)	2282 (60.2%)	1508 (39.8%)		
**History of Prematurity**					
*Yes*	214 (5.3%)	119 (55.6%)	95 (44.4%)	0.86 (0.65–1.13)	0.27
*No*	3818 (94.7%)	2256 (59.4%)	1542 (40.6%)		
**Underlying Conditions**					
*Yes*	843 (20.9%)	425 (50.4%)	418 (49.6%)	0.64 (0.56–0.74)	0.001
*No*	3189 (79.1%)	1960 (61.5%)	1229 (38.5%)		

Data are presented by median (IQR: Q1–Q3) or number (%). IQR: Interquartile Range. * A *p*-value less than 0.05 is statistically significant. COVID-19: Coronavirus disease 2019.

**Table 3:** The differences in Anti-SARS-CoV-2 antibody titer levels among seropositive subgroups.

Subgroups	Positive, N = 2385, n (%)	Mean (UI/mL)	Median (UI/mL)	IQR	*p*-Value *
**Age *(months)***					
*≤12*	556 (23.3%)	12.9	5.6	2.6–10.4	<0.001
*13 to <36*	642 (26.9%)	10.5	5.7	3.1–10.1	
*36 to <60*	361 (15.1%)	13.0	4.1	1.9–7.7	
*60 to <144*	750 (31.5%)	12.8	3.4	1.9–7.7	
*≥144*	76 (3.2%)	165.7	63.3	7.8–257.9	
**Gender**					
*Male*	1434 (60.1%)	18.3	4.9	2.4–9.7	0.36
*Female*	951 (39.9%)	15.3	4.6	2.4–9.1	
**History of Prematurity**					
*Yes*	119 (5.0%)	16.5	4.1	2.6–9.4	0.90
*No*	2256 (94.6%)	17.1	4.8	2.4–9.6	
**Residences**					
*Hanoi*	1540 (64.6%)	18.9	4.9	2.4–9.6	0.07
*Other provinces*	845 (35.4%)	13.8	4.5	2.4–9.2	
**Types of Patient Care**					
*In-patient*	59 (2.5%)	30.0	7.9	2.7–16.4	*0.01*
*Out-patient*	2326 (97.5%)	16.8	4.7	2.4–9.4	
**Previous COVID-19 Infection of Household Members**					
*Yes*	1630 (68.3%)	13.2	4.6	2.4–8.9	*0.02*
*No*	755 (31.7%)	25.5	5.2	2.4–12.1	
**History of Using Corticosteroids or Immunosuppressants**					
*Yes*	103 (4.3%)	15.2	3.5	1.9–9.8	0.10
*No*	2282 (95.7%)	17.2	4.8	2.4–9.6	
**Underlying Conditions**					
*Yes*	425 (17.8%)	17.6	3.8	2.1–8.5	*0.005*
*No*	1960 (82.1%)	17.0	4.9	2.4–9.7	

Data are presented by median (IQR: Q1–Q3) or number (%). IQR: Interquartile Range. * A *p*-value less than 0.05 is statistically significant. COVID-19: Coronavirus disease 2019.

**Table 4:** Multivariate logistic regression for independent factors associated with seropositivity (R-squared = 4.69%).

Variables	OR	Estimate with 95% CI	*p*-Value *
Age group			
*≤12 months*	1		
*13 to < 36 months*	1.29	1.06–1.56	0.01
*36 to < 60 months*	0.94	0.76–1.63	0.58
*60 to < 144 months*	0.79	0.67–0.95	0.01
*≥144 months*	1.84	1.21–2.80	0.005
Residence of Hanoi	1.54	1.34–1.77	<0.001
Presence of COVID-19 infection of household members	2.36	2.06–2.70	<0.001
Presence of underlying condtions	0.71	0.60–0.85	<0.001
History of using corticosteroids or immunosuppressants	0.64	0.48–0.86	0.003

OR: Odds ratio; CI: Confidence Interval. * A *p*-value less than 0.05 is statistically significant. COVID-19: Coronavirus disease 2019.

**Table 5:** Influence of maternal history during pregnancy/labor period in seropositivity of Anti-SARS-CoV-2 antibodies for 939 infants aged ≤ 12 months.

Variables	Positive, N (%)	Negative, N (%)	OR (95%CI)	*p*-Value *
**Maternal History of COVID-19 Vaccination**				
*Yes*	276 (60.5%)	180 (39.5%)	1.11 (0.85–1.45)	0.42
*No*	280 (58.0%)	203 (42.0%)		
**Maternal History of COVID-19 Infection**				
*Yes*	224 (70.9%)	92 (29.1%)	2,06 (1.54–2.75)	0.0001
*No*	335 (53.8%)	288 (46.2%)		
**Breastfeeding Status**				
*Yes*	495 (60.1%)	328 (39.9%)	1.36 (0.90–2.04)	0.12
*No*	61 (52.6%)	55 (47.4%)		

OR: Odds ratio; CI: Confidence Interval. * A *p*-value less than 0.05 is statistically significant. COVID-19: Coronavirus disease 2019.

## Data Availability

All data are available upon request.

## References

[1] Zhu N., Zhang D., Wang W., Li X., Yang B., Song J., Zhao X., Huang B., Shi W., Lu R. (2020). A Novel Coronavirus from Patients with Pneumonia in China, 2019. N. Engl. J. Med..

[2] Hu B., Guo H., Zhou P., Shi Z.L. (2021). Characteristics of SARS-CoV-2 and COVID-19. Nat. Rev. Microbiol..

[3] Huang C., Wang Y., Li X., Ren L., Zhao J., Hu Y., Zhang L., Fan G., Xu J., Gu X. (2020). Clinical features of patients infected with 2019 novel coronavirus in Wuhan, China. Lancet.

[4] Mehta O.P., Bhandari P., Raut A., Kacimi S.E.O., Huy N.T. (2021). Coronavirus Disease (COVID-19): Comprehensive Review of Clinical Presentation. Front. Public Health.

[5] Gao Y., Chen Y., Liu M., Shi S., Tian J. (2020). Impacts of immunosuppression and immunodeficiency on COVID-19: A systematic review and meta-analysis. J. Infect..

[6] Sanyaolu A., Okorie C., Marinkovic A., Patidar R., Younis K., Desai P., Hosein Z., Padda I., Mangat J., Altaf M. (2020). Comorbidity and its Impact on Patients with COVID-19. SN Compr. Clin. Med..

[7] Mehta N.S., Mytton O.T., Mullins E., Fowler T.A., Falconer C.L., Murphy O.B., Langenberg C., Jayatunga W., Eddy D.H., Nguyen Van Tam J.S. (2020). SARS-CoV-2 (COVID-19): What Do We Know About Children? A Systematic Review. Clin. Infect. Dis..

[8] Chou J., Thomas P.G., Randolph A.G. (2022). Immunology of SARS-CoV-2 infection in children. Nat. Immunol..

[9] Viner R.M., Mytton O.T., Bonell C., Melendez-Torres G.J., Ward J., Hudson L., Waddington C., Thomas J., Russell S., van der Klis F. (2021). Susceptibility to SARS-CoV-2 Infection Among Children and Adolescents Compared With Adults: A Systematic Review and Meta-analysis. JAMA Pediatr..

[10] Principi N., Bosis S., Esposito S. (2010). Effects of coronavirus infections in children. Emerg. Infect. Dis..

[11] Wu Z., McGoogan J.M. (2020). Characteristics of and Important Lessons From the Coronavirus Disease 2019 (COVID-19) Outbreak in China: Summary of a Report of 72,314 Cases From the Chinese Center for Disease Control and Prevention. JAMA.

[12] Levorson R.E., Christian E., Hunter B., Sayal J., Sun J., Bruce S.A., Garofalo S., Southerland M., Ho S., Levy S. (2021). A cross-sectional investigation of SARS-CoV-2 seroprevalence and associated risk factors in children and adolescents in the United States. PLoS ONE.

[13] Dawood F.S., Porucznik C.A., Veguilla V., Stanford J.B., Duque J., Rolfes M.A., Dixon A., Thind P., Hacker E., Castro M. (2022). Incidence Rates, Household Infection Risk, and Clinical Characteristics of SARS-CoV-2 Infection Among Children and Adults in Utah and New York City, New York. JAMA Pediatr..

[14] Wachter F., Regensburger A.P., Antonia S.P., Knieling F., Wagner A.L., Simon D., Hoerning A., Woelfle J., Überla K., Neubert A. (2022). Continuous monitoring of SARS-CoV-2 seroprevalence in children using residual blood samples from routine clinical chemistry. Clin. Chem. Lab. Med..

[15] Dong Y., Mo X., Hu Y., Qi X., Jiang F., Jiang Z., Tong S. (2020). Epidemiology of COVID-19 Among Children in China. Pediatrics.

[16] Alfraij A., Bin A.A.A., Al-Otaibi A.M., Alsharrah D., Aldaithan A., Kamel A.M., Almutairi M., Alshammari S., Almazyad M., Macarambon J.M. (2021). Characteristics and outcomes of coronavirus disease 2019 (COVID-19) in critically ill pediatric patients admitted to the intensive care unit: A multicenter retrospective cohort study. J. Infect. Public Health.

[17] Takahashi H., Ichinose N., Okada Y. (2022). False-negative rate of SARS-CoV-2 RT-PCR tests and its relationship to test timing and illness severity: A case series. IDCases.

[18] Kanji J.N., Zelyas N., MacDonald C., Pabbaraju K., Khan M.N., Prasad A., Hu J., Diggle M., Berenger B.M., Tipples G. (2021). False negative rate of COVID-19 PCR testing: A discordant testing analysis. Virol. J..

[19] Waterfield T., Watson C., Moore R., Ferris K., Tonry C., Watt A., McGinn C., Foster S., Evans J., Lyttle M.D. (2021). Seroprevalence of SARS-CoV-2 antibodies in children: A prospective multicentre cohort study. Arch. Dis. Child..

[20] Long Q.X., Liu B.Z., Deng H.J., Wu G.C., Deng K., Chen Y.K., Liao P., Qiu J.F., Lin Y., Cai X.F. (2020). Antibody responses to SARS-CoV-2 in patients with COVID-19. Nat. Med..

[21] Sethuraman N., Jeremiah S.S., Ryo A. (2020). Interpreting Diagnostic Tests for SARS-CoV-2. JAMA.

[22] Zhang X., Lu S., Li H., Wang Y., Lu Z., Liu Z., Lai Q., Ji Y., Huang X., Li Y. (2020). Viral and Antibody Kinetics of COVID-19 Patients with Different Disease Severities in Acute and Convalescent Phases: A 6-Month Follow-Up Study. Virol. Sin..

[23] McConnell D., Hickey C., Bargary N., Trela-Larsen L., Walsh C., Barry M., Adams R. (2021). Understanding the Challenges and Uncertainties of Seroprevalence Studies for SARS-CoV-2. Int. J. Environ. Res. Public Health.

[24] Lewis H.C., Ware H., Whelan M., Subissi L., Li Z., Ma X., Nardone A., Valenciano M., Cheng B., Noel K. (2022). SARS-CoV-2 infection in Africa: A systematic review and meta-analysis of standardised seroprevalence studies, from January 2020 to December 2021. BMJ Glob. Health.

[25] Vial P., González C., Icaza G., Ramirez-Santana M., Quezada-Gaete R., Núñez-Franz L., Apablaza M., Vial C., Rubilar P., Correa J. (2022). Seroprevalence, spatial distribution, and social determinants of SARS-CoV-2 in three urban centers of Chile. BMC Infect. Dis..

[26] ADVIA Centaur SARS-CoV-2 Total (COV2T)—Instructions for Use. https://www.fda.gov/media/138446/download.

[27] Sinaei R., Pezeshki S., Parvaresh S., Sinaei R. (2021). Why COVID-19 is less frequent and severe in children: A narrative review. World J. Pediatr..

[28] Posfay-Barbe K.M., Wagner N., Gauthey M., Moussaoui D., Loevy N., Diana A., L’Huillier A.G. (2020). COVID-19 in Children and the Dynamics of Infection in Families. Pediatrics.

[29] Gudbjartsson D.F., Helgason A., Jonsson H., Magnusson O.T., Melsted P., Norddahl G.L., Saemundsdottir J., Sigurdsson A., Sulem P., Agustsdottir A.B. (2020). Spread of SARS-CoV-2 in the Icelandic Population. N. Engl. J. Med..

[30] Dimeglio C., Mansuy J.M., Charpentier S., Claudet I., Izopet J. (2020). Children are protected against SARS-CoV-2 infection. J. Clin. Virol..

[31] Stringhini S., Wisniak A., Piumatti G., Azman A.S., Lauer S.A., Baysson H., De Ridder D., Petrovic D., Schrempft S., Marcus K. (2020). Seroprevalence of anti-SARS-CoV-2 IgG antibodies in Geneva, Switzerland (SEROCoV-POP): A population-based study. Lancet.

[32] Knabl L., Mitra T., Kimpel J., Rössler A., Volland A., Walser A., Ulmer H., Pipperger L., Binder S.C., Riepler L. (2021). High SARS-CoV-2 seroprevalence in children and adults in the Austrian ski resort of Ischgl. Commun. Med. (Lond.).

[33] Lenicek K.J., Zrinski T.R., Stevanovic V., Lukic-Grlic A., Tabain I., Misak Z., Roic G., Kaic B., Mayer D., Hruskar Z. (2021). Seroprevalence of SARS-CoV-2 infection among children in Children’s Hospital Zagreb during the initial and second wave of COVID-19 pandemic in Croatia. Biochem. Med. (Zagreb).

[34] Zinszer K., McKinnon B., Bourque N., Pierce L., Saucier A., Otis A., Cheriet I., Papenburg J., Hamelin M.È., Charlana K. (2021). Seroprevalence of SARS-CoV-2 Antibodies Among Children in School and Day Care in Montreal, Canada. JAMA Netw. Open.

[35] Callaway E. (2022). Why does the Omicron sub-variant spread faster than the original?. Nature.

[36] Madhi S.A., Kwatra G., Myers J.E., Jassat W., Dhar N., Mukendi C.K., Nana A.J., Blumberg L., Welch R., Ngorima-Mabhena N. (2022). Population Immunity and Covid-19 Severity with Omicron Variant in South Africa. N. Engl. J. Med..

[37] Kundu A., Maji S., Kumar S., Bhattacharya S., Chakraborty P., Sarkar J. (2022). Clinical aspects and presumed etiology of multisystem inflammatory syndrome in children (MIS-C): A review. Clin. Epidemiol. Glob. Health.

[38] Mallapaty S. (2022). COVID jabs for kids: They’re safe and they work—So why is uptake so patchy?. Nature.

[39] Anderson E.J., Campbell J.D., Creech C.B., Frenck R., Kamidani S., Munoz F.M., Nachman S., Spearman P. (2021). Warp Speed for Coronavirus Disease 2019 (COVID-19) Vaccines: Why Are Children Stuck in Neutral?. Clin. Infect. Dis..

[40] Saxena S., Skirrow H., Wighton K. (2022). Vaccinating children aged under 5 years against COVID-19. BMJ.

[41] Lv M., Luo X., Shen Q., Lei R., Liu X., Liu E., Li Q., Chen Y. (2021). Safety, Immunogenicity, and Efficacy of COVID-19 Vaccines in Children and Adolescents: A Systematic Review. Vaccines.

[42] Tian F., Yang R., Chen Z. (2022). Safety and efficacy of COVID-19 vaccines in children and adolescents: A systematic review of randomized controlled trials. J. Med. Virol..

[43] Pollán M., Pérez-Gómez B., Pastor-Barriuso R., Oteo J., Hernán M.A., Pérez-Olmeda M., Sanmartín J.L., Fernández-García A., Cruz I., Fernández de Larrea N. (2020). Prevalence of SARS-CoV-2 in Spain (ENE-COVID): A nationwide, population-based seroepidemiological study. Lancet.

[44] Wu Q., Xing Y., Shi L., Li W., Gao Y., Pan S., Wang Y., Wang W., Xing Q. (2020). Coinfection and Other Clinical Characteristics of COVID-19 in Children. Pediatrics.

[45] Ismail S.A., Saliba V., Lopez Bernal J., Ramsay M.E., Ladhani S.N. (2021). SARS-CoV-2 infection and transmission in educational settings: A prospective, cross-sectional analysis of infection clusters and outbreaks in England. Lancet Infect. Dis..

[46] Callender L.A., Curran M., Bates S.M., Mairesse M., Weigandt J., Betts C.J. (2020). The Impact of Pre-existing Comorbidities and Therapeutic Interventions on COVID-19. Front. Immunol..

[47] Zilla M.L., Keetch C., Mitchell G., McBreen J., Shurin M.R., Wheeler S.E. (2021). SARS-CoV-2 Serologic Immune Response in Exogenously Immunosuppressed Patients. J. Appl. Lab. Med..

[48] Pietrasanta C., Ronchi A., Crippa B.L., Artieri G., Ballerini C., Crimi R., Mosca F., Pugni L. (2022). Coronavirus Disease 2019 Vaccination During Pregnancy and Breastfeeding: A Review of Evidence and Current Recommendations in Europe, North America, and Australasia. Front. Pediatr..

[49] Rad H.S., Röhl J., Stylianou N., Allenby M.C., Bazaz S.R., Warkiani M.E., Guimaraes F., Clifton V.L., Kulasinghe A. (2021). The Effects of COVID-19 on the Placenta During Pregnancy. Front. Immunol..

[50] Juan J., Gil M.M., Rong Z., Zhang Y., Yang H., Poon L.C. (2020). Effect of coronavirus disease 2019 (COVID-19) on maternal, perinatal and neonatal outcome: Systematic review. Ultrasound Obstet. Gynecol..

[51] Ng K.W., Faulkner N., Cornish G.H., Rosa A., Harvey R., Hussain S., Ulferts R., Earl C., Wrobel A.G., Benton D.J. (2020). Preexisting and de novo humoral immunity to SARS-CoV-2 in humans. Science.

